# Supplementary material for: Inhibition of microbial biofuel production in drought-stressed switchgrass hydrolysate
Source: Biotechnol Biofuels. 2016 Nov 8;9:237. doi: 10.1186/s13068-016-0657-0 (PMC5100259; doi:10.1186/s13068-016-0657-0)
Supplement: Supplementary file 2 — Additional file 2. Maps of significant gene ontology terms for chemical genomics data. Untreated biomass composition. Detailed hydrolysate composition. [file 13068_2016_657_MOESM2_ESM.pdf]

Supporting Information

Reaction products of drought stressed bioenergy crops inhibit microbial biofuel production

Rebecca Garlock Ong[a,b,c1], Alan Higbee[d], Scott Bottoms[e], Quinn Dickinson[e], Dan Xie[e], Scott A. Smith[f], Jose Serate[e], Edward Pohlmann[e], Arthur Daniel Jones[f,g,h], Joshua J. Coon[d,i,j], Trey K. Sato[e], Gregg R. Sanford[e,k], Dustin Eilert[e], Lawrence G. Oates[e,k], Jeff S. Piotrowski[e], Donna M. Bates[e], David Cavalier[a], and Yaoping Zhang[e]

[a]DOE Great Lakes Bioenergy Research Center, Michigan State University, East Lansing, MI;
[b]Department of Chemical Engineering, Michigan State University, East Lansing, MI;
[c]Department of Chemical Engineering, Michigan Technological University, Houghton, MI;
[d]Department of Chemistry, University of Wisconsin-Madison, Madison, WI;
[e]DOE Great Lakes Bioenergy Research Center, University of Wisconsin-Madison, Madison, WI;
[f]RTSF Mass Spectrometry & Metabolomics Core, Michigan State University, East Lansing, MI
[g]Department of Biochemistry & Molecular Biology, Michigan State University, East Lansing, MI
[h]Department of Chemistry, Michigan State University, East Lansing, MI
[i]Department of Biomolecular Chemistry, University of Wisconsin-Madison, Madison, WI
[j]Genome Center of Wisconsin, University of Wisconsin-Madison, Madison, WI
[k]Department of Agronomy, University of Wisconsin-Madison, Madison, WI

Contents

Fig. S1: Enriched Gene Ontology (GO) terms for highly susceptible yeast mutants in all hydrolysates. 2

Fig. S2: Enriched GO terms for select hydrolysates. 3

Table S1: Untreated biomass composition (% of total dry biomass). 4

Table S2: Hydrolysate composition data: Carbohydrates, organic acids, and aromatics. 5

Table S3: Hydrolysate composition data: Metals and inorganics. 7

Table S4: Hydrolysate composition data: Amino acids. 8

Fig. S1: Enriched Gene Ontology (GO) terms for highly susceptible yeast mutants in all hydrolysates. Susceptible mutants were chosen that had a fitness <-2.5 (n=65). P-values were Bonferroni corrected with a cutoff of p=0.05.

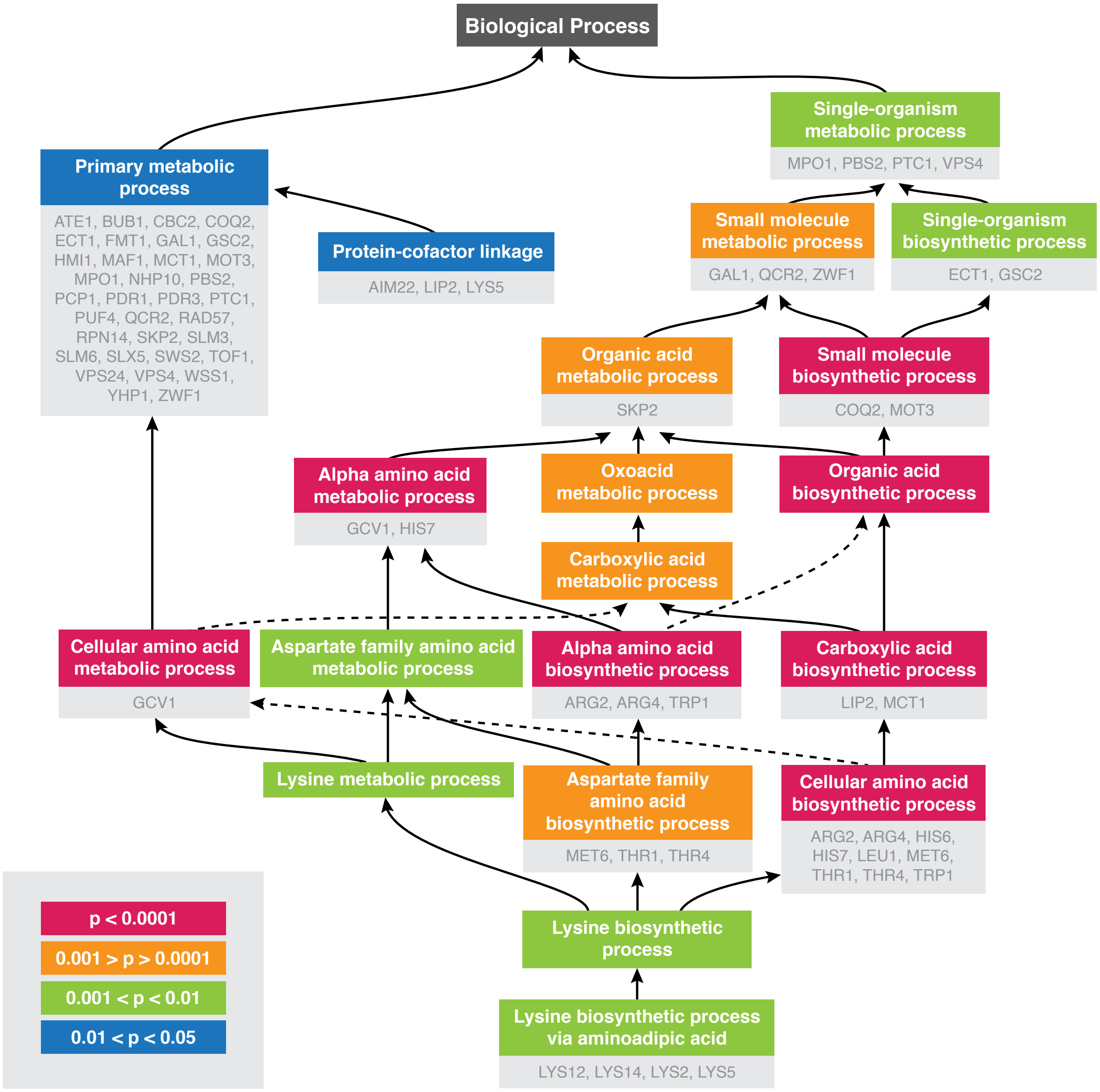

Fig. S2: Enriched GO terms for select hydrolysates. A) Enriched GO terms For highly susceptible yeast mutants in hydrolysates 2010 ACSH, 2012 ACSH-PO448R, 2013 ACSH, and 2013 ASGH; Mutants were chosen that had fitness <-2.5 in all four hydrolysates and >-2.5 in 2012 ASGH, and were statistically different between the two groups (p<0.001, n=56). P-values for the GO terms were Bonferroni corrected with a cutoff of p = 0.05. **B)** Enriched GO terms for highly resistant yeast mutants in only 2012 ASGH. Mutants were chosen that had fitness >2.5 in the 2012 ASGH, <2.5 in the other four hydrolysates and a statistically significant difference between the two groups (p<0.001, n=42). P-values for the GO terms were Bonferroni corrected with a cutoff of p=0.05.

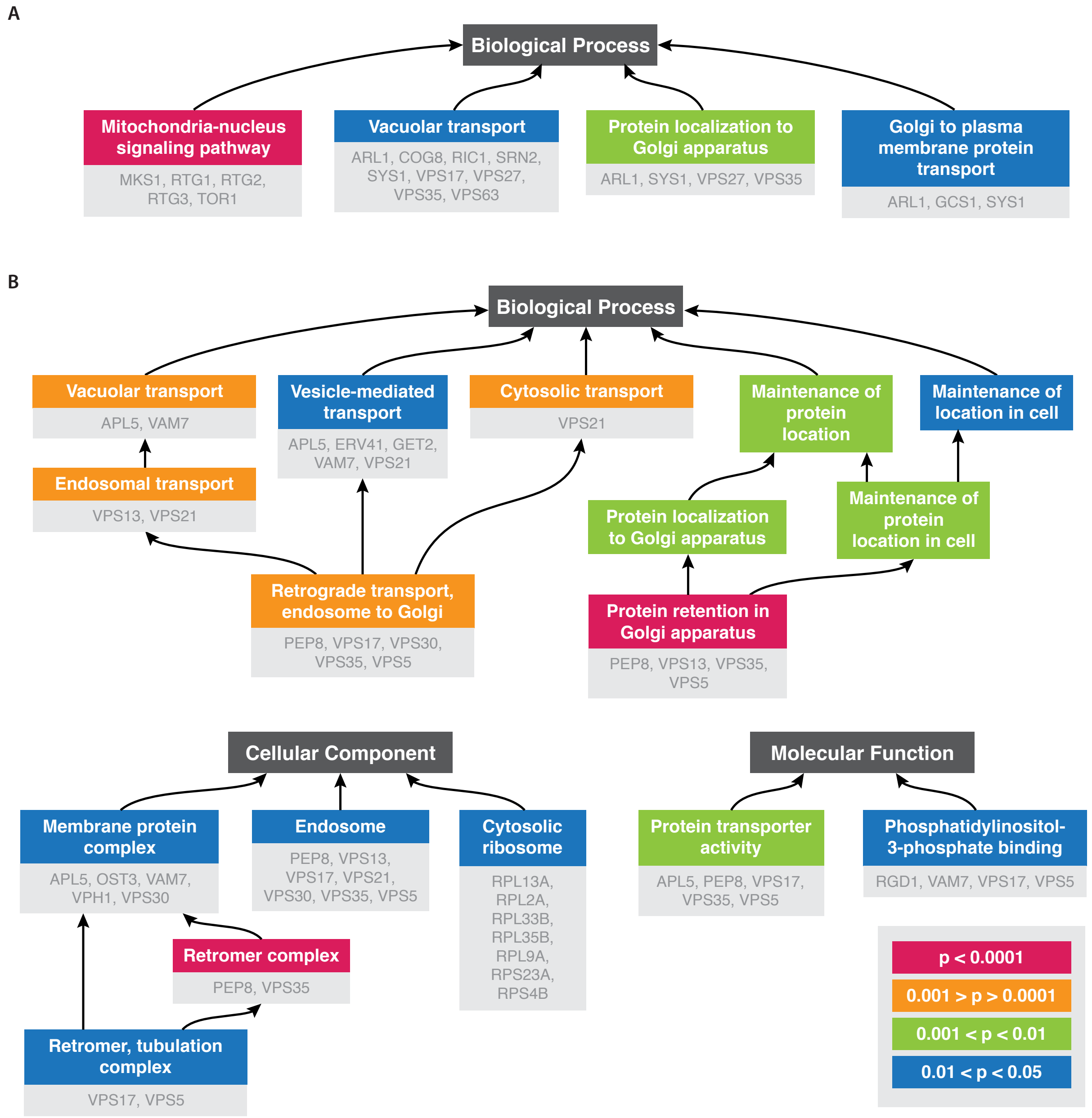

Tables

Table S1: Untreated biomass composition (% of total dry biomass). Values are reported as the mean ± s.d. (n=3).

		Corn Stover				Switchgrass		
		36H56		P0448R		Shawnee		CIR
		2010	2012	2012	2013	2010	2012	2013
**Total Ash**		13.39 ± 0.16	6.48 ± 0.09	4.75 ± 0.30	5.26 ± 0.04	5.56 ± 0.16	5.47 ± 0.08	4.68 ± 0.04
**Total Extractives**		16.00 ± 0.04	15.29 ± 0.09	18.87 ± 0.11	16.51 ± 0.36	14.90 ± 0.26	22.14 ± 0.21	10.86 ± 0.65
**Water Extractives**	**Sucrose**	0.57 ± 0.02	0.08 ± 0.01	0.00 ± 0.00	0.00 ± 0.00	0.43 ± 0.01	2.40 ± 0.07	0.14 ± 0.02
	**Fructose**	1.11 ± 0.04	0.88 ± 0.01	2.25 ± 0.04	1.80 ± 0.02	0.51 ± 0.01	2.33 ± 0.02	0.33 ± 0.01
	**Glucose**	0.93 ± 0.03	1.18 ± 0.04	2.04 ± 0.04	0.95 ± 0.01	0.62 ± 0.01	2.57 ± 0.03	0.61 ± 0.03
	**Xylose**	0.00 ± 0.00	0.16 ± 0.01	0.08 ± 0.00	0.03 ± 0.01	ND	0.04 ± 0.00	0.01 ± 0.01
	**Galactose**	0.20 ± 0.00	0.00 ± 0.00	0.04 ± 0.00	0.02 ± 0.00	ND	0.06 ± 0.00	0.03 ± 0.00
	**Arabinose**	0.00 ± 0.00	0.04 ± 0.00	0.06 ± 0.00	0.04 ± 0.00	ND	0.04 ± 0.00	0.00 ± 0.01
	**Mannose**	NM*	NM	ND†	ND	NM	ND	ND
	**Glucan**	0.75 ± 0.01	0.44 ± 0.01	1.05 ± 0.04	0.67 ± 0.01	0.62 ± 0.02	0.98 ± 0.38	0.49 ± 0.02
	**Xylan**	0.25 ± 0.00	0.20 ± 0.01	0.26 ± 0.00	0.22 ± 0.01	0.06 ± 0.00	0.07 ± 0.01	0.08 ± 0.01
	**Galactan**	0.19 ± 0.00	0.22 ± 0.01	0.24 ± 0.00	0.20 ± 0.01	0.05 ± 0.00	0.31 ± 0.02	0.19 ± 0.01
	**Arabinan**	0.17 ± 0.01	0.12 ± 0.01	0.12 ± 0.01	0.09 ± 0.01	0.18 ± 0.01	0.07 ± 0.01	0.05 ± 0.01
	**Mannan**	NM	NM	0.17 ± 0.01	0.09 ± 0.01	NM	ND	ND
**Ethanol Extractives**		2.59 ± 0.06	2.95 ± 0.06	2.85 ± 0.15	2.66 ± 0.01	2.08 ± 0.19	2.61 ± 0.03	1.97 ± 0.08
**Extracted Protein**		1.74 ± 0.07	2.50 ± 0.04	2.40 ± 0.17	1.25 ± 0.08	1.16 ± 0.09	1.89 ± 0.41	0.85 ± 0.18
**Total Protein**		4.79 ± 0.07	6.29 ± 0.01	5.55 ± 0.15	2.93 ± 0.08	3.19 ± 0.09	4.65 ± 0.08	2.63 ± 0.18
**Total Structural Sugars‡**		53.43 ± 0.21	58.04 ± 0.34	56.86 ± 0.28	61.57 ± 0.14	59.50 ± 0.18	52.28 ± 0.09	62.49 ± 0.27
**Glucan**		30.67 ± 0.16	30.98 ± 0.32	31.74 ± 0.24	36.47 ± 0.10	34.24 ± 0.11	29.36 ± 0.07	35.90 ± 0.23
**Xylan**		18.47 ± 0.13	20.88 ± 0.10	20.49 ± 0.16	21.41 ± 0.09	21.54 ± 0.13	18.91 ± 0.04	23.15 ± 0.13
**Galactan**		1.17 ± 0.05	2.57 ± 0.03	1.47 ± 0.01	1.07 ± 0.01	1.43 ± 0.03	1.31 ± 0.02	0.98 ± 0.03
**Arabinan**		3.12 ± 0.02	3.62 ± 0.01	3.16 ± 0.02	2.62 ± 0.02	2.30 ± 0.03	2.69 ± 0.01	2.45 ± 0.05
**Mannan**		NM	NM	ND	ND	NM	ND	ND
**Acetyl Groups**		2.22 ± 0.06	2.62 ± 0.00	2.38 ± 0.31	2.91 ± 0.15	2.58 ± 0.11	2.15 ± 0.06	2.38 ± 0.20
**Total Lignin**		14.31 ± 0.03	14.70 ± 0.26	14.39 ± 0.18	14.76 ± 0.02	18.59 ± 0.11	15.38 ± 0.06	18.95 ± 0.12
**Acid Insoluble Lignin**		13.08 ± 0.02	13.18 ± 0.26	13.13 ± 0.16	13.55 ± 0.02	17.87 ± 0.10	14.31 ± 0.06	17.94 ± 0.11
**Acid Soluble Lignin**		1.23 ± 0.01	1.52 ± 0.04	1.27 ± 0.07	1.21 ± 0.01	0.73 ± 0.03	1.07 ± 0.01	1.01 ± 0.03
**Mass Balance Closure**		102.39	100.93	100.40	102.69	102.44	100.17	101.13

*NM = not measured
†ND = not detected
‡Total structural sugars does not include acetyl groups

Table S2: Hydrolysate composition data: Carbohydrates, organic acids, and aromatics. Values are reported as the mean ± s.d. (n=3). In each row, values with different superscripts are statistically different based on Tukey's HSD Test (95% CI).

	Corn Stover				Switchgrass		
	36H56		P0448R		Shawnee		CIR
	2010	2012	2012	2013	2010	2012	2013
Major Carbohydrates, Acids, and Amides (mM)							
Glucose	355.5^{abc} ± 7.9	355.0^{bc} ± 5.8	369.2^{ab} ± 5.6	375.1^{a} ± 11.3	328.7^{d} ± 4.2	334.8^{cd} ± 11.4	329.8^{d} ± 4.3
Xylose	182.2^{c} ± 2.5	211.2^{ab} ± 3.6	192.5^{bc} ± 11.6	204.1^{bc} ± 11.1	208.0^{bc} ± 2.5	204.5^{bc} ± 16.6	235.3^{a} ± 9.1
Glycerol	7.0^{a} ± 2.5	5.5^{abc} ± 0.7	6.2^{ab} ± 0.2	4.0^{bc} ± 0.6	3.6^{bc} ± 0.4	4.7^{abc} ± 0.1	3.0^{c} ± 0.2
Formate	3.6^{c} ± 0.6	2.8^{c} ± 2.4	5.0^{bc} ± 0.4	5.3^{bc} ± 1.5	7.6^{ab} ± 2.0	11.3^{a} ± 0.6	8.5^{ab} ± 0.5
Lactate	0.1^{c} ± 0.0	1.4^{b} ± 0.1	1.7^{a} ± 0.0	0.0^{c} ± 0.0	0.0^{c} ± 0.0	0.1^{c} ± 0.0	0.0^{c} ± 0.0
Succinate	1.0^{bc} ± 0.0	0.6^{cd} ± 0.1	1.4^{a} ± 0.3	1.0^{b} ± 0.2	0.4^{d} ± 0.1	0.6^{cd} ± 0.0	0.9^{bc} ± 0.1
Acetate	31.3^{c} ± 2.7	31.4^{c} ± 2.7	36.5^{bc} ± 3.0	33.9^{c} ± 1.5	42.1^{ab} ± 1.6	46.7^{a} ± 3.4	42.9^{ab} ± 2.2
Acetamide	77.6 ± 8.6	71.1 ± 29.5	82.8 ± 10.2	83.1 ± 10.2	88.9 ± 2.5	113.7 ± 32.6	89.8 ± 17.1
Coumaric Acid	1.3^{a} ± 0.1	0.5^{c} ± 0.1	0.8^{b} ± 0.1	0.9^{b} ± 0.1	0.2^{d} ± 0.0	0.3^{cd} ± 0.0	0.3^{cd} ± 0.0
Coumaroyl Amide	5.0^{a} ± 0.6	3.2^{c} ± 0.4	3.8^{bc} ± 0.3	4.5^{ab} ± 0.6	1.6^{d} ± 0.4	1.8^{d} ± 0.3	1.9^{d} ± 0.4
Feruloyl Amide	2.0^{a} ± 0.1	1.5^{b} ± 0.2	2.2^{a} ± 0.1	2.2^{a} ± 0.2	0.6^{d} ± 0.1	1.2^{bc} ± 0.2	0.8^{cd} ± 0.2
Aliphatic and Aromatic Acids (mM)							
Azelaic Acid	24.1^{b} ± 0.3	21.4^{bc} ± 0.9	27.9^{a} ± 1.0	18.0^{d} ± 1.5	19.1^{cd} ± 0.8	21.9^{bc} ± 1.4	17.7^{d} ± 1.1
Benzoic Acid	132.3^{c} ± 8.3	168.2^{bc} ± 39.7	121.3^{c} ± 5.3	123.7^{c} ± 38.4	304.5^{a} ± 2.8	224.6^{b} ± 6.0	299.9^{a} ± 8.2
3-Hydroxybenzoic Acid	0.5^{a} ± 0.1	0.2^{b} ± 0.1	0.6^{a} ± 0.1	0.7^{a} ± 0.1	0.6^{a} ± 0.2	0.6^{a} ± 0.0	0.7^{a} ± 0.0
4-Hydroxybenzoic Acid	28.1^{bc} ± 3.2	48.1^{a} ± 10.1	21.2^{c} ± 0.5	18.0^{c} ± 2.0	36.1^{ab} ± 6.0	18.2^{c} ± 1.0	18.5^{c} ± 1.5
2,3-Dihydroxybenzoic Acid	<1.75*^{c}	NM	<1.75*^{c}	<1.75*^{c}	NM	13.1^{b} ± 0.4	18.5^{a} ± 0.6
3,4-Dihydroxybenzoic Acid	5.8^{cd} ± 2.2	4.6^{d} ± 1.2	6.2^{cd} ± 0.5	5.9^{cd} ± 0.8	10.3^{c} ± 3.5	15.7^{b} ± 0.5	20.9^{a} ± 1.2
Ferulic Acid	39.1^{a} ± 1.9	25.2^{bc} ± 4.8	29.2^{ab} ± 3.5	37.7^{a} ± 7.9	16.5^{c} ± 1.2	18.5^{bc} ± 2.1	19.6^{bc} ± 2.7
Sinapic Acid	3.0^{a} ± 0.3	1.2^{b} ± 0.2	2.3^{a} ± 04	0.7*^{bc} ± 0.6	0.2*^{c} ± 0.0	1.0^{b} ± 0.2	0.8^{bc} ± 0.0
Syringic Acid	11.2 ± 0.9	9.6 ± 1.4	10.7 ± 0.7	10.1 ± 1.2	9.4 ± 1.8	9.1 ± 0.5	10.4 ± 0.2
Vanillic Acid	26.7^{d} ± 2.5	29.0^{cd} ± 4.2	26.5^{d} ± 1.9	19.5^{d} ± 2.6	51.5^{a} ± 9.6	38.9^{bc} ± 2.5	43.1^{ab} ± 0.2
Aromatic Alcohols (mM)							
Furfuryl Alcohol	5.6^{ab} ± 1.1	<3*^{b}	7.0^{ab} ± 1.8	5.1*^{ab} ± 3.6	2.1*^{b} ± 0.0	7.5^{a} ± 1.6	2.8*^{ab} ± 2.5
4-Hydroxybenzyl Alcohol	0.4^{bc} ± 0.0	<0.15*^{c}	0.3^{c} ± 0.0	0.4^{bc} ± 0.1	0.4*^{bc} ± 0.5	1.1^{a} ± 0.0	0.8^{ab} ± 0.1
Vanillyl Alcohol	<0.25*^{d}	0.3^{c} ± 0.0	0.3^{c} ± 0.0	<0.25*^{d}	0.6^{b} ± 0.0	0.8^{a} ± 0.0	0.6^{b} ± 0.0
Aromatic Aldehydes (mM)							
Furfural	10.5^{b} ± 1.7	41.1^{a} ± 3.9	37.5^{a} ± 6.6	29.8^{ab} ± 10.5	44.3^{a} ± 18.0	8.9^{b} ± 4.2	27.5^{ab} ± 4.0
HMF	0.9 ± 0.3	1.1 ± 0.3	1.2 ± 0.1	1.0 ± 0.1	1.0 ± 0.3	1.3 ± 0.2	1.3 ± 0.0
4-Hydroxybenzaldehyde	31.7^{a} ± 4.0	21.6^{ab} ± 3.5	17.5^{bc} ± 2.5	31.1^{a} ± 7.0	18.3^{b} ± 5.7	6.0^{c} ± 0.5	16.1^{bc} ± 2.5
Syringaldehyde	10.3^{ab} ± 0.8	7.4^{abc} ± 1.7	8.5^{abc} ± 1.0	11.2^{a} ± 3.1	5.1^{c} ± 2.3	3.8^{c} ± 1.2	6.1^{bc} ± 1.0
Vanillin	180.8^{a} ± 13.5	141.5^{a} ± 14.5	156.4^{a} ± 13.0	145.8^{a} ± 28.9	71.5^{b} ± 26.5	69.4^{b} ± 12.8	85.8^{b} ± 13.4
Aromatic Amides (mM)							
4-Hydroxybenzamide	18.5^{a} ± 5.0	12.0^{bc} ± 1.3	15.4^{ab} ± 1.3	10.5^{bc} ± 0.9	9.6^{bc} ± 2.2	8.0^{c} ± 0.7	12.8^{abc} ± 1.2
Benzamide	3.4^{d} ± 0.6	3.1^{d} ± 0.3	2.4^{d} ± 0.2	2.0^{d} ± 0.2	6.5^{b} ± 1.0	10.0^{a} ± 0.3	4.9^{c} ± 0.3
Syringamide	36.9^{ab} ± 10.1	42.5^{a} ± 11.1	29.8^{ab} ± 1.4	32.6^{ab} ± 4.6	27.0^{ab} ± 6.6	21.3^{b} ± 2.2	35.3^{ab} ± 2.7
Vanillamide	78.3^{ab} ± 17.0	108.0^{a} ± 31.7	73.8^{ab} ± 4.5	57.5^{b} ± 5.5	69.9^{ab} ± 20.6	64.6^{ab} ± 3.3	71.3^{ab} ± 7.7
Aromatic Ketones (mM)							
4-Hydroxyacetophenone	1.7 ± 0.2	2.0 ± 0.6	2.2 ± 0.7	2.4 ± 0.6	2.2 ± 0.8	1.2 ± 0.1	2.3 ± 0.4
Acetovanillone	3.0^{b} ± 0.5	28.1^{a} ± 21.6	4.3^{b} ± 0.9	3.5^{b} ± 0.6	25.3^{ab} ± 6.1	3.7^{b} ± 0.3	4.2^{b} ± 0.5
Acetosyringone	4.3^{ab} ± 0.8	0.2*^{c} ± 0.1	3.7^{ab} ± 1.0	4.8^{a} ± 0.9	1.0^{c} ± 0.3	3.3^{ab} ± 0.2	2.9^{b} ± 0.2
Diferulates (mM)							
8-O-4-Diferulic Acid	1.1^{b} ± 0.3	1.4^{b} ± 0.2	2.4^{a} ± 0.6	1.4^{b} ± 0.3	0.9^{b} ± 0.2	0.9^{b} ± 0.0	1.1^{b} ± 0.3
8,5'-Diferulic Acid	0.6^{ab} ± 0.0	0.3^{bc} ± 0.1	0.6^{a} ± 0.1	0.3^{c} ± 0.1	0.1*^{c} ± 0.1	0.2^{c} ± 0.0	0.2*^{c} ± 0.1
8,8'-Diferulic Acid	2.6^{bcd} ± 0.2	3.9^{ab} ± 1.0	5.1^{a} ± 0.5	3.6^{abc} ± 0.5	2.0^{cd} ± 1.0	1.5^{d} ± 0.0	2.0^{cd} ± 0.4

*One or more replicates were below the limit of detection (LOD). Where all replicates were below the limit, the LOD is reported with no standard deviation. When fewer than all were below the limit, the values were recalculated as LOD/√2 [52]. These recalculated values were used to determine the mean, standard deviation, and statistical differences.

Table S3: Hydrolysate composition data: Metals and inorganics. Values are reported as the mean ± s.d. (n=3). In each row, values with different superscripts are statistically different based on Tukey's HSD Test (95% CI).

	Corn Stover				Switchgrass		
	36H56		P0448R		Shawnee		CIR
	2010	2012	2012	2013	2010	2012	2013
Abundant Metals and Inorganics (mM)							
Total N	237.8^{cd} ± 7.0	252.7^{bc} ± 6.8	263.6^{b} ± 3.8	223.2^{d} ± 10.5	226.4^{d} ± 8.7	307.3^{a} ± 3.8	236.9^{cd} ± 1.2
NH_4-N	39.4^{cd} ± 1.7	49.7^{abc} ± 3.8	42.1^{bcd} ± 0.7	33.0^{d} ± 1.5	51.2^{ab} ± 9.0	56.2^{a} ± 1.4	43.5^{bc} ± 1.9
K	41.3^{b} ± 6.9	39.6^{b} ± 1.0	44.4^{b} ± 0.8	41.4^{b} ± 1.4	52.3^{a} ± 1.6	39.9^{b} ± 1.8	26.3^{c} ± 0.6
P	11.4^{c} ± 0.5	17.4^{a} ± 1.0	11.0^{c} ± 0.1	8.6^{d} ± 1.1	12.5^{c} ± 0.9	14.8^{b} ± 0.6	15.2^{ab} ± 0.6
Mg	19.3^{b} ± 0.1	11.7^{d} ± 0.3	10.5^{d} ± 0.1	8.4^{e} ± 0.4	14.8^{c} ± 0.8	24.4^{a} ± 1.0	15.1^{c} ± 0.5
Ca	7.2^{a} ± 0.5	4.5^{b} ± 0.2	3.5^{c} ± 0.0	3.4^{c} ± 0.5	0.6^{d} ± 0.0	6.4^{a} ± 0.2	0.8^{d} ± 0.0
S	3.9^{de} ± 0.2	4.3^{cd} ± 0.2	3.6^{e} ± 0.1	2.8^{f} ± 0.2	4.8^{b} ± 0.1	5.4^{a} ± 0.2	4.6^{bc} ± 0.1
Na	2.4^{e} ± 0.1	1.7^{f} ± 0.0	2.3^{e} ± 0.0	2.7^{d} ± 0.1	3.0^{c} ± 0.1	3.7^{b} ± 0.1	4.4^{a} ± 0.1
Cl†	63.8^{c} ± 11.9	59.3^{c} ± 3.6	66.6^{bc} ± 3.3	64.3^{c} ± 0.8	80.6^{b} ± 1.1	96.6^{a} ± 5.1	56.6^{c} ± 2.0
Trace Metals and Inorganics (mM)							
Al	25.8^{a} ± 10.8	9.9^{b} ± 3.6	<3.7*^{b}	3.9*^{b} ± 2.6	8.8*^{b} ± 5.4	<3.7*^{b}	<3.7*^{b}
B	<3.7*^{c}	19.1^{a} ± 2.8	<3.7*^{c}	<3.7*^{c}	6.8^{b} ±	<3.7*^{c}	<3.7*^{c}
Co	0.5^{a} ± 0.2	<0.1*^{b}	<0.1*^{b}	0.2^{b} ± 0.0	<0.1*^{b}	<0.1*^{b}	<0.1*^{b}
Cr	6.6^{ab} ± 5.6	4.8^{ab} ± 0.7	0.4^{b} ± 0.0	6.9^{a} ± 0.7	5.4^{ab} ± 1.2	2.8^{ab} ± 2.6	1.4^{ab} ± 0.3
Cu	1.3^{ab} ± 1.4	2.5^{a} ± 0.7	1.6^{ab} ± 0.2	0.5*^{b} ± 0.4	0.9^{ab} ± 0.1	1.4^{ab} ± 0.2	0.4^{b} ± 0.1
Fe	358.1^{b} ± 202.8	23.8^{c} ± 3.5	14.9^{c} ± 4.2	1234.5^{a} ± 176.1	16.9^{c} ± 2.4	23.0^{c} ± 5.2	4.4^{c} ± 1.4
Ni	2.7 ± 3.4	2.6 ± 0.3	0.3 ± 0.0	0.1* ± 0.0	1.4 ± 0.4	0.9 ± 0.7	0.2 ± 0.1
Li	1.4^{b} ± 0.2	0.4*^{c} ± 0.3	0.8^{bc} ± 0.1	2.9^{a} ± 0.1	0.4*^{c} ± 0.3	3.2^{a} ± 0.3	0.7^{c} ± 0.0
Mn	195.5^{a} ± 11.1	88.8^{c} ± 14.1	56.4^{d} ± 3.4	136.7^{b} ± 9.1	38.9^{d} ± 3.5	45.9^{d} ± 5.3	34.3^{d} ± 1.7
Mo	1.1^{a} ± 0.4	0.3^{b} ± 0.1	<0.1*^{b}	0.9^{a} ± 0.1	0.3^{b} ± 0.1	0.3^{b} ± 0.1	0.1*^{b} ± 0.1
Zn	16.8^{a} ± 1.7	15.6^{a} ± 0.6	9.9^{bc} ± 1.1	16.0^{a} ± 2.1	7.1^{cd} ± 0.2	11.4^{b} ± 1.1	4.7^{d} ± 0.3

*One or more replicates were below the limit of detection (LOD). Where all replicates were below the limit, the LOD is reported with no standard deviation. When fewer than all were below the limit, the values were recalculated as LOD/√2 [52]. These recalculated values were used to determine the mean, standard deviation, and statistical differences.

Table S4: Hydrolysate composition data: Amino acids. Values are reported as the mean ± s.d. (n=3). In each row, values with different superscripts are statistically different based on Tukey's HSD Test (95% CI).

	Corn Stover				Switchgrass		
	36H56		P0448R		Shawnee		CIR
	2010	2012	2012	2013	2010	2012	2013
Amino Acids* (mM)							
Glycine	723 ± 123	740 ± 64	657 ± 72	828 ± 43	586 ± 145	754 ± 54	642 ± 156
Alanine	759^{b} ± 237	1391^{a} ± 143	1327^{a} ± 118	650^{b} ± 49	643^{b} ± 56	1236^{a} ± 74	558^{b} ± 71
Valine	174^{d} ± 25	462^{a} ± 25	331^{c} ± 14	143^{d} ± 11	153^{d} ± 5	388^{b} ± 27	168^{d} ± 14
Leucine	259^{cd} ± 19	392^{a} ± 12	320^{b} ± 13	180^{e} ± 9	227^{de} ± 34	387^{a} ± 11	281^{bc} ± 11
Isoleucine	97^{c} ± 11	241^{a} ± 5	183^{b} ± 10	77^{c} ± 1	77^{c} ± 8	178^{b} ± 10	87^{c} ± 3
Phenylalanine	175^{bc} ± 8	198^{ab} ± 6	182^{bc} ± 8	110^{d} ± 2	159^{c} ± 19	226^{a} ± 9	185^{bc} ± 17
Tryptophan	17^{cd} ± 3	26^{b} ± 4	25^{b} ± 1	12^{d} ± 1	21^{bc} ± 3	43^{a} ± 4	23^{bc} ± 1
Proline	357^{c} ± 55	980^{b} ± 39	1045^{b} ± 18	116^{d} ± 8	373^{c} ± 22	1465^{a} ± 86	138^{d} ± 21
Serine	310^{c} ± 16	442^{ab} ± 59	359^{bc} ± 9	258^{c} ± 35	281^{c} ± 90	565^{a} ± 18	312^{c} ± 32
Threonine	750^{bc} ± 67	1543^{a} ± 95	1158^{ab} ± 95	546^{c} ± 38	884^{bc} ± 137	1115^{b} ± 143	754^{bc} ± 170
Tyrosine	168^{bcd} ± 26	250^{a} ± 9	204^{abc} ± 24	113^{d} ± 13	137^{cd} ± 41	211^{ab} ± 15	193^{abc} ± 38
Asparagine	476^{a} ± 145	495^{a} ± 144	384^{ab} ± 54	196^{b} ± 49	434^{ab} ± 95	352^{ab} ± 47	232^{ab} ± 103
Lysine	192^{b} ± 31	303^{ab} ± 29	223^{b} ± 10	144^{b} ± 19	159^{b} ± 28	475^{a} ± 174	177^{b} ± 29
Arginine	200^{b} ± 27	214^{ab} ± 21	174^{b} ± 38	111^{b} ± 26	176^{b} ± 22	415^{a} ± 192	118^{b} ± 15
Histidine	49^{bc} ± 6	88^{a} ± 9	66^{abc} ± 8	42^{c} ± 2	55^{bc} ± 12	71^{ab} ± 15	55^{bc} ± 7
Aspartic Acid	856^{c} ± 85	1414^{a} ± 17	1093^{b} ± 61	384^{e} ± 31	578^{d} ± 32	708^{cd} ± 100	554^{d} ± 31
Glutamic Acid	413^{cd} ± 47	879^{a} ± 15	726^{b} ± 15	308^{e} ± 33	341^{de} ± 20	343^{de} ± 54	439^{c} ± 11

*The amino acids cysteine and methionine are unable to be quantified in hydrolysate samples using the analytical method. Although glutamine could be quantified, it was not observed for most samples. This amino acid is notoriously unstable and may have degraded during storage.

References

52. Croghan C, Egeghy PP. Methods of dealing with values below the limit of detection using SAS. Southern SAS User Group; St. Petersburg, FL; 2003.
